# COVID-19 symptoms associated with smoking and vaping tobacco and cannabis: A cross-sectional analysis

**DOI:** 10.1016/j.pmedr.2025.103291

**Published:** 2025-10-30

**Authors:** Sifon U. Ndon, Andrew W. Liu, Timothy J. Judson, Pamela M. Ling

**Affiliations:** a Department of Otolaryngology-Head and Neck Surgery, University of California San Francisco, San Francisco, CA 94143, USA.; b School of Medicine, University of Massachusetts Chan Medical School, Worcester, MA 01655, USA.; c Department of Medicine, University of California, San Francisco, San Francisco, CA 94143, USA; d Office of Population Health, University of California, San Francisco, San Francisco, CA 94143, USA; e Center for Tobacco Control Research and Education, University of California, San Francisco, San Francisco, CA 94143, USA.

**Keywords:** Smoking, Tobacco use, Vaping, cannabis, Respiratory tract infections, Risk factors, Substance-related disorders

## Abstract

**Objective:**

Characterize symptoms observed among people using nicotine and/or cannabis compared to non-users using an institutional respiratory illness symptom checker.

**Methods:**

Cross-sectional study (April 2020–August 2021) using University of California San Francisco (UCSF) COVID-19 & Flu Symptom Checker online self-triage tool measuring current tobacco and cannabis use, symptom prevalence and associations with self-reported severe dyspnea, using multivariable logistic regression.

**Results:**

Excluding those with emergent symptoms requiring immediate medical evaluation, of 10,904 patients, 729 (6.7%) self-reported current nicotine or cannabis use. People smoking and/or vaporizing nicotine or cannabis reported constitutional, ear/nose and throat, respiratory, and gastrointestinal symptoms more frequently than non-users. Current vaping (aOR = 1.89, 95% CI 1.23, 2.81) and co-use of nicotine/cannabis (aOR = 2.00, 95% CI 1.26, 3.09) were associated with higher odds of dyspnea, adjusting for comorbidities and demographic factors.

**Conclusion:**

People using nicotine or cannabis products had increased frequencies of self-reported COVID-19 symptoms and dyspnea. Current vaporizer use may not be associated with a difference in self-reported respiratory symptoms compared to smoking tobacco. Including tobacco and cannabis use in symptom checkers may enhance risk stratification and clinical decision-making during future respiratory virus outbreaks.

## Highlights

• Tobacco and cannabis use is rarely included in respiratory symptom checkers
• Both smoking and vaping modes of use were associated with severe symptoms (dyspnea)
• Co-use of both nicotine and cannabis was associated with dyspnea
• Using vaporizers for nicotine or cannabis may not decrease respiratory symptoms
• Including smoking/vaping status may improve digital triage for respiratory illness

## 1 Introduction

Use of e-cigarettes and cannabis products is increasingly common, and research on the health impacts is needed. Approximately 5% of adults self-reported e-cigarette use in 2021 (Kramarow and Elgaddal, 2023), and e-cigarettes are the most common tobacco product used by young adults and adolescents. As nicotine e-cigarette use (colloquially called “vaping”) rose in recent years, cannabis vaping increased in parallel (Kramarow and Elgaddal, 2023), with cannabis vaping more than doubling among both adolescents and young adults (National Institute on Drug Abuse, 2020). Dual use of e-cigarettes and cannabis is more common than use of e-cigarettes alone (Moustafa et al., 2022).

As cannabis use is legalized in an increasing number of states, cannabis use and exposure to cannabis smoke are increasingly perceived as safe (Bhatia et al., 2022). However, studies have found that combustion of cannabis, whether through smoking or vaping, produces a greater amount of particulate matter than tobacco, raising concerns that it could have adverse health consequences (Glantz et al., 2018). Co-use may present even greater toxicological exposure risks (Smith et al., 2019). People who use both tobacco and cannabis are more likely to use tobacco (and cannabis) products heavily and have greater nicotine dependence (Akbar et al., 2019), exhibit problem behaviors related to cannabis use, and have poorer cessation outcomes (Vogel et al., 2018) than single product users. Co-use of tobacco and cannabis is associated with greater depression and anxiety than use of tobacco or cannabis alone (Nguyen et al., 2023). While co-use has been reported to be associated with additive psychiatric, psychosocial, mental health disorders, and toxicological risks (Ramo et al., 2012), few studies address health symptoms among co-users, particularly including both smoking and vaporized tobacco and cannabis products.

Studies of respiratory symptoms and vaping are largely limited to single product use, and most focus on adolescents and young adults. For example, e-cigarette use was associated with symptoms of bronchitis and shortness of breath in a study of adolescents and young adults (Chaffee et al., 2021). A meta-analysis of cannabis smoking and respiratory symptoms found that compared with nonsmokers, cannabis users had an increased risk of cough, sputum production, wheezing, and dyspnea (Ghasemiesfe et al., 2018). Few studies have addressed cannabis vaping, and most are limited to young populations: one study of adolescents found greater asthma symptoms among those using cannabis in e-cigarettes (Boyd et al., 2021), and a study of young adults found cannabis vaping was associated with bronchitis symptoms and wheezing (Braymiller et al., 2020).

During the COVID-19 pandemic, respiratory symptom screeners were widely used to assess and triage large numbers of patients. Smoking status and/or use of vaporized nicotine and cannabis is largely not accounted for within symptom checkers. However, there may be an important interplay between patients' smoking and/or e-cigarette or vaporizer use and their symptomatology. The University of California San Francisco (UCSF) COVID-19 & Flu Symptom Checker includes measures of both smoked and vaporized nicotine and cannabis use, allowing us to examine (1) What COVID-19 related symptoms do people who smoke and use vaporizers for nicotine and cannabis report? and (2) Are there differences in severe symptoms (dyspnea) by mode of product use (smoking versus vaporizer use) or type (nicotine, cannabis or co-use)? We performed a descriptive analysis of symptom checker data characterizing the constellation of symptoms seen among people using tobacco or cannabis by smoking and/or vaporizing compared to people who did not use, and tested associations between nicotine and cannabis product form and type with severe dyspnea.

## 2 Methods

### 2.1 Study and population

A cross-sectional study was carried out using data collected from the UCSF COVID-19 & Flu Symptom Checker developed for use by UCSF patients with symptoms of an upper respiratory infection (Judson et al., 2020). The COVID-19 & Flu Symptom Checker, launched in March 2020, is an electronic medical record-associated self-triage and self-scheduling tool for individuals with symptoms of upper respiratory infections. The tool asked patients to answer a series of branched logic questions regarding exposure history, comorbidities, and symptoms in order to risk-stratify and direct them to the appropriate level of care. This tool was modified in April 2020 to include questions regarding tobacco and cannabis smoking and vaporizer use. This tool was available in Spanish and English to all adults ≥ 18 years of age with an active patient portal. The same algorithm was used for those who sought guidance via the institutional COVID-19 telephone hotline. Individuals who had unique clinical circumstances (i.e., 20 or more weeks pregnant) or reported emergent symptoms requiring immediate medical evaluation (i.e., chest pain, severe shortness of breath with associated cyanosis or difficulty speaking, confusion, seizures, coughing up blood, and slurred speech) were directed to specialty triage or to the Emergency Department before completing the symptom checker.

A total of 11,865 patients started the symptom checker, and 961 patients had emergent symptoms necessitating acute evaluation prior to completing the symptom checker. These patients did not provide information on comorbidities and smoking/vaping status, and were excluded from analysis. There were 10,904 patients who completed the symptom checker including the smoking/vaping status questions between April 2020 and August 2021. All data was self-reported, and included age, sex, race/ethnicity, insurance status, preferred language, COVID-19 exposure history, symptoms, comorbidities, and tobacco and cannabis use history. All questions were yes/no questions (e.g., having symptoms) or “select all that apply.” All questions were mandatory, except for the circumstances specified above. Null responses were treated as missing data and thus were excluded from the dataset for analysis, yielding a final analytic sample (*N* = 10,670) for multivariable analysis. COVID-19 test results were documented for those who scheduled a test after using the symptom checker.

### 2.2 Measures

#### 2.2.1 Outcome variables

We performed descriptive analyses with demographic characteristics, COVID-19 symptoms, and medical comorbidities as outcomes. Demographics included self-reported gender, race/ethnicity, age, preferred language and type of health insurance. COVID-19 symptoms were assessed as yes/no questions, including constitutional (e.g. fatigue, fever), neurologic (e.g. headache), head eyes ears nose and throat (e.g., congestion), respiratory (e.g., cough), and gastrointestinal (e.g. diarrhea). For multivariable analyses, the primary outcome was new or worsening dyspnea, which was the best proxy for significant respiratory symptoms that did not qualify for emergency triage. The other respiratory symptom (cough) commonly follows respiratory infections and does not necessarily indicate more severe disease. For the primary outcome, the symptom screener was worded in layman's terms to capture new onset or worsening dyspnea: “Are you having trouble breathing that is unusual for you? For example, are you having to stop and catch your breath more than usual while walking or going up stairs?”

#### 2.2.2 Exposure variables

To determine smoking/vaping status, all individuals were asked, “Do you currently smoke or vape?” and those who indicated “yes” were asked to indicate which of the following they regularly do, selecting all that apply from 5 choices: smoke cigarettes, vape nicotine (e-cigarettes), vape marijuana, smoke marijuana, or “other smoking/vaping product”. Individuals who reported smoking or vaping were characterized as “users,” with those who denied use of any of these substances classified as “non-users.” Individuals who reported use of more than one type of any smoking or vaping product were classified as “multiple product users.” We also classified individuals by type of product (nicotine, cannabis, dual-user, or non-user). Patients who reported “other smoking/vaping product” were excluded. Due to question wording, the time frame of current smoking or vaping (e.g. past 30 days) was not specified. Mild to moderate symptoms were included in this analysis because emergent symptoms were triaged directly to the emergency department.

#### 2.2.3 Covariates

Covariates included in multivariate modeling included demographics (age, sex, race/ethnicity, insurance status), as well as medical comorbidities. Medical comorbidities were self-reported yes/no questions for having any conditions that weaken the immune system (cancer being actively treated, any organ transplant or bone marrow transplant, any condition for which you are currently taking steroid pills or any other therapy targeting the immune system, HIV with CD4 lymphocyte count below 200, or any other diagnosed immune system problem), or being told by a doctor that they had chronic lung disease (asthma, COPD, emphysema or other lung disease), congestive heart failure or a weak heart, chronic kidney disease requiring dialysis, cirrhosis, hypertension, and severe obesity BMI ≥40.

### 2.3 Statistical analysis

Symptoms and comorbidities were treated as binary variables, and users of nicotine and/or cannabis and non-users were compared using chi-squared tests. Two multivariable logistic regression models were performed using dyspnea as the primary outcome. The models controlled for demographic characteristics and medical comorbidities excluding cases with missing data. The first model included mode of use (smoking, vaporizer, dual-user, non-user), while the second model included product type (nicotine, cannabis, dual-user, non-user). Variable inflation factors were calculated to assess collinearity as model diagnostics with a cutoff <10 for model inclusion. Comorbidity covariates were defined (e.g., BMI ≥40) based on clinical relevance to immune dysfunction, dyspnea, and highest risk of severe COVID-19. A sensitivity analysis was conducted excluding patients with chronic lung disease. All analyses were conducted in R 3.5.1; a *p*-value <0.05 was considered significant.

The study was approved by the UCSF Institutional Review Board (IRB). All data collection was carried out in accordance with the IRB data safety specifications.

## 3 Results

### 3.1 Demographic characteristics of tobacco and cannabis users

Of the 10,904 patients included in the analysis, 6.7% (*N* = 729) self-identified as current users of tobacco or cannabis (Table 1). There were 193 users reporting use of multiple nicotine or cannabis products (26.5% of current users). Among those who reported use, there were more female respondents, and the majority were White and English speakers. Users of nicotine or cannabis were younger than non-users and more likely to be male, non-white, and non-commercially insured compared to non-users. Approximately 5% (*N* = 542) of all patients ultimately tested positive for COVID-19 at UCSF.

**Table 1:** Descriptive statistics of demographics of adult patients completing COVID-19 & Flu Symptom Checker (April 2020–August 2021) by tobacco and cannabis use status.

	**Cigarette Smoking** **N (%)**	**Cannabis Smoking** **N (%)**	**Cannabis Vaporizer Use N(%)**	**Nicotine Vaporizer Use N(%)**	**Multiple Product Use N (%)**	**Non-Use N(%)**	**P-Value**⁎
Number of Responses (*n* = 10,904)	205	163	91	77	193	10,175	
Age							<0.01
18–29 (*n* = 2050)	21 (10.2)	70 (42.9)	32 (35.2)	30 (39.0)	87 (45.1)	1810 (17.8)	
30–49 (*n* = 5601)	114 (55.6)	66 (40.5)	34 (37.4)	36 (46.8)	81 (42.0)	5270 (51.8)	
50–59 (*n* = 1467)	41 (20.0)	18 (11.0)	9 (9.9)	6 (7.8)	14 (7.3)	1379 (13.6)	
60+ (*n* = 1786)	29 (14.1)	9 (5.5)	16 (17.6)	5 (6.5)	11 (5.7)	1716 (16.9)	
Gender							<0.01
Male (*n* = 3623)	80 (39.0)	67 (41.1)	48 (52.7)	30 (39.0)	84 (43.5)	3314 (32.6)	
Female (*n* = 7254)	125 (61.0)	95 (58.3)	41 (45.1)	47 (61.0)	107 (55.4)	6839 (67.2)	
Nonbinary (n = 14)	0 (0.0)	1 (0.6)	2 (2.2)	0 (0.0)	2 (1.0)	9 (0.1)	
Unknown (*n* = 13)	0 (0.0)	0 (0.0)	0 (0.0)	0 (0.0)	0 (0.0)	13 (0.1)	
Race/Ethnicity							<0.01
White (*n* = 6005)	104 (50.7)	77 (47.2)	60 (65.9)	35 (45.5)	85 (44.0)	5644 (55.5)	
Black (*n* = 428)	16 (7.8)	29 (17.8)	3 (3.3)	2 (2.6)	21 (10.9)	357 (3.5)	
Hispanic or Latino (*n* = 1378)	25 (12.2)	23 (14.1)	12 (13.2)	15 (19.5)	40 (20.7)	1263 (12.4)	
Asian American, Native Hawaiian/Pacific Islander (*n* = 1964)	33 (16.1)	9 (5.5)	4 (4.4)	12 (15.6)	17 (8.8)	1889 (18.6)	
Other (*n* = 1129)	27 (13.2)	25 (15.3)	12 (13.2)	13 (16.9)	30 (15.5)	1022 (10.0)	
Preferred Language							0.74
English (n = 10,783)	201 (98.0)	162 (99.4)	90 (98.9)	76 (98.7)	192 (99.5)	10,062 (98.9)	
Spanish (*n* = 45)	0 (0.0)	0 (0.0)	0 (0.0)	0 (0.0)	0 (0.0)	45 (0.4)	
Chinese (*n* = 23)	1 (0.5)	0 (0.0)	0 (0.0)	0 (0.0)	0 (0.0)	22 (0.2)	
Other (*n* = 53)	3 (1.5)	1 (0.6)	1 (1.1)	1 (1.3)	1 (0.5)	46 (0.5)	
Insurance							<0.01
Commercial (*n* = 7446)	104 (50.7)	106 (65.0)	59 (64.8)	51 (66.2)	111 (57.5)	7015 (68.9)	
Medicare (*n* = 1205)	31 (15.1)	12 (7.4)	13 (14.3)	9 (11.7)	18 (9.3)	1122 (11.0)	
Medicaid (*n* = 981)	57 (27.8)	30 (18.4)	7 (7.7)	6 (7.8)	55 (28.5)	826 (8.1)	
Other (*n* = 1038)	12 (5.9)	10 (6.1)	8 (8.8)	7 (9.1)	6 (3.1)	995 (9.8)	

⁎ p-value based on chi-square tests.

### 3.2 COVID-19 symptoms by tobacco and cannabis use

The most prevalent symptoms reported among non-users were fatigue, sore throat, sinus congestion and headache (Table 2). Users of nicotine and/or cannabis products demonstrated a similar pattern of symptoms, and overall nicotine and cannabis users reported symptoms more frequently than non-users; these included muscle aches, chills, fever, weakness/dizziness, loss of taste or smell, any dyspnea, diarrhea, abdominal pain, nausea/vomiting, and difficulty keeping down fluids.

**Table 2:** Descriptive statistics of mild and moderate symptoms reported by adult patients on COVID-19 & Flu symptom checker (April 2020–August 2021) by tobacco and cannabis use status.

	**Cigarette Smoking N(%)**	**Cannabis Smoking N(%)**	**Cannabis Vaporizer Use N(%)**	**Nicotine Vaporizer Use N(%)**	**Multiple Product Use N(%)**	**Non-Use N(%)**	**P-Value**⁎
Number of Responses (n = 10,904)	205	163	91	77	193	10,175	
Constitutional symptoms							
Fatigue (*n* = 5997)	107 (52.2)	97 (59.5)	57 (62.6)	46 (59.7)	109 (56.5)	5581 (54.9)	0.41
Muscle Aches (*n* = 3222)	74 (36.1)	53 (32.5)	33 (36.3)	36 (46.8)	73 (37.8)	2953 (29.0)	<0.01
Chills (*n* = 2734)	64 (31.2)	44 (27.0)	26 (28.6)	22 (28.6)	67 (34.7)	2515 (24.7)	0.04
Fever (*n* = 1795)	43 (21.0)	33 (20.2)	16 (17.6)	11 (14.3)	28 (14.5)	1664 (16.4)	<0.01
Weak/Dizzy (*n* = 614)	23 (11.2)	20 (12.3)	9 (9.9)	7 (9.1)	20 (10.4)	535 (5.3)	<0.01
Neurologic							
Headache (*n* = 5795)	113 (55 0.1)	85 (52.1)	51 (56.0)	45 (58.4)	102 (52.8)	5399 (53.1)	0.90
Head, eyes, ears, nose and throat							
Sore Throat (*n* = 5935)	95 (46.3)	85 (52.1)	48 (52.7)	50 (64.9)	104 (53.9)	5553 (54.6)	0.09
Sinus Congestion (*n* = 5802)	116 (56.6)	101 (62.0)	50 (54.9)	42 (54.5)	113 (58.5)	5380 (52.9)	0.12
Loss of Taste/Smell (*n* = 576)	19 (9.3)	16 (9.8)	7 (7.7)	11 (14.3)	28 (14.5)	495 (4.9)	<0.01
Conjunctivitis (*n* = 203)	4 (2.0)	3 (1.8)	4 (4.4)	1 (1.3)	3 (1.6)	188 (1.8)	0.63
Respiratory							
Ongoing Cough (*n* = 3729)	65 (31.7)	66 (40.5)	31 (34.1)	25 (32.5)	71 (36.8)	3471 (34.1)	0.53
Trouble Breathing (Dyspnea) (*n* = 1113)	29 (14.1)	21 (12.9)	16 (17.6)	10 (13.0)	33 (17.1)	1004 (9.9)	<0.01
Gastrointestinal							
Diarrhea (*n* = 1713)	30 (14.6)	37 (22.7)	23 (25.3)	17 (22.1)	53 (27.5)	1553 (15.3)	<0.01
Abdominal Pain (*n* = 1492)	41 (20.0)	25 (15.3)	19 (20.9)	18 (23.3)	43 (22.3)	1346 (13.2)	<0.01
Vomiting (*n* = 559)	10 (4.9)	28 (17.2)	13 (14.3)	4 (5.2)	26 (13.5)	478 (4.7)	<0.01
Difficulty keeping down fluids (*n* = 89)	3 (4.9)	6 (3.7)	1 (1.1)	0 (0.0)	4 (2.1)	75 (0.7)	<0.01

⁎ p-value based on Chi-square tests.

### 3.3 Comorbidities

Those who completed the Symptom Checker were asked to report the presence of medical comorbidities that have been shown in the literature to place individuals at higher risk for worse COVID-19-related outcomes (e.g., need for hospital admission, admission to the intensive care unit, need for mechanical ventilation, death) (Sanchez-Ramirez and Mackey, 2020; Mahamat-Saleh et al., 2021; Richardson et al., 2020; Zheng et al., 2020). The comorbidity reported most frequently among respondents was chronic lung disease (14.7%), followed by hypertension (11.2%) (Table 3). Overall, nicotine and cannabis users self-reported chronic lung disease and hypertension more frequently than non-users.

**Table 3:** Respondent self-reported comorbidities in patients completing symptom checker (April 2020–August 2021) by nicotine and cannabis use.

	**Cigarette Smoking (n = 205)**	**Cannabis Smoking (*n* = 163)**	**Cannabis Vaporizer Use (*n* = 91)**	**Nicotine Vaporizer Use (*n* = 77)**	**Multiple Product Use (*n* = 193)**	**Non-Use (n = 10,175)**
Immunocompromised (*n* = 1136)	36 (17.6)	17 (10.4)	11 (12.1)	5 (6.5)	23 (11.9)	1044 (10.3)
Chronic Lung Disease (*n* = 1608)	43 (21.0)	32 (19.6)	13 (14.3)	11 (14.3)	36 (18.7)	1473 (14.5)
Congestive Heart Failure (*n* = 127)	4 (2.0)	0 (0.0)	0 (0.0)	1 (1.3)	6 (3.1)	116 (1.1)
Diabetes (*n* = 430)	15 (7.3)	7 (4.3)	4 (4.4)	3 (3.9)	9 (4.7)	392 (3.9)
CKD Requiring Dialysis (*n* = 41)	1 (0.5)	1 (0.6)	0 (0.0)	0 (0.0)	1 (0.5)	38 (0.4)
Cirrhosis (n = 41)	3 (1.5)	2 (1.2)	0 (0.0)	0 (0.0)	0 (0.0)	36 (0.4)
Hypertension (*n* = 1218)	42 (20.5)	19 (11.7)	10 (11.0)	9 (11.7)	26 (13.5)	1112 (10.9)
Obesity (BMI ≥ 40) (*n* = 316)	8 (3.9)	7 (4.3)	4 (4.4)	6 (7.8)	4 (2.1)	287 (2.8)
Comorbidities per patient reported (mean/IQR)	0.7 ± 0.9	0.5 ± 0.8	0.5 ± 0.8	0.5 ± 0.8	0.5 ± 0.9	0.5 ± 0.7

CKD: Chronic Kidney Disease.

BMI: Body Mass Index.

**Table 4:** Mode of tobacco or cannabis product use and associations with dyspnea in multivariable logistic regression models controlling for demographics and comorbidities (n = 10,670).

Characteristic	aOR⁎	95% CI
Smoking or Vaping Tobacco or Cannabis products (vs non-User)		
Current Exclusive Smoking	1.29	0.94, 1.73
Current Exclusive Vaporizer Use	1.89	1.23, 2.81
Current Smoking and Vaping	2.00	1.26, 3.09

⁎ aOR adjusted for age, sex, race/ethnicity, insurance status, and comorbidities (immunocompromised, chronic lung disease, heart failure, chronic kidney disease, diabetes, cirrhosis, hypertension and obesity).

**Table 5:** Factors associated with dyspnea in multivariable logistic regression model of smoking product types, demographics, and comorbidities (n = 10,670).⁎

Characteristic	aOR⁎	95% CI
Active Product Status (vs Non-User)		
Current Exclusive Nicotine use	1.41	0.99, 1.96
Current Exclusive Cannabis use	1.56	1.11, 2.14
Current Nicotine and Cannabis use	2.01	1.17, 3.29

⁎ aOR adjusted for age, sex, race/ethnicity, insurance status and listed comorbidities (immunocompromised, chronic lung disease, heart failure, chronic kidney disease, diabetes, cirrhosis, hypertension and obesity).

### 3.4 Factors associated with dyspnea

We conducted two multivariable logistic regression models with patient reported dyspnea as the primary outcome. We found that active vaporizing of nicotine or cannabis and co-use was associated with dyspnea (Table 4). Using either tobacco or cannabis exclusively via vaporizers (aOR = 1.89, 95% CI 1.23, 2.81) or reporting both smoking and vaping either product (aOR = 2.00, 95% CI 1.26, 3.09) were associated with increased odds of dyspnea compared to non-users, controlling for demographics and comorbidities. Exclusively smoking tobacco or cannabis was associated with dyspnea in univariate analyses (OR = 1.39 95% CI 1.03, 1.85) (Supplementary Table 1), but this association was no longer independently associated when controlling for demographics and comorbidities (aOR = 1.29, 95% CI 0.94, 1.73). While the analysis in Table 4 focused on mode of use (smoked, vaped or both) for either nicotine or cannabis products, when nicotine and cannabis use were entered as separate predictors (both smoking and vaporized) in models, both were also found to be independently associated with higher odds of reporting dyspnea, for exclusive nicotine (aOR = 1.41, 95% CI 0.99, 1.96), exclusive cannabis (aOR = 1.56, 95% CI 1.112, 2.14) and use of both nicotine and cannabis (aOR = 2.01, 95% CI 1.17, 3.29) (Table 5). The associations remained significant in a sensitivity analysis excluding patients with chronic lung disease, with the exception of exclusive nicotine use, which was of borderline statistical significance (Supplemental Table 2).

## 4 Discussion

In this study of more than 10,000 outpatients seeking triage for respiratory symptoms, active nicotine, cannabis, or multiple product use was independently associated with a higher likelihood of reporting dyspnea, even after accounting for chronic disease status. Overall, active users of nicotine or cannabis products reported a similar constellation of mild and moderate symptoms as non-users, but at higher rates. Active users were also more likely to report having comorbid chronic conditions, which may partially explain increased symptom rates. Prior studies have shown the presence of dyspnea to be a predictor of severe COVID-19 infection and disease progression (Shi et al., 2020; Zheng et al., 2020). These findings are consistent with existing literature, which demonstrates an association between cigarette smoking and increased upper respiratory infection symptomatology (Benowitz et al., 2022; Zhou et al., 2018). This may in part be due to the baseline increased frequency of symptoms, particularly respiratory symptoms, among those who smoke and vaporize nicotine products compared to their non-user counterparts (Sargent et al., 2022; Varella et al., 2022). The increased symptomatology may also be partially explained by the increased rates of chronic disease among users.

Respiratory illness symptom checkers generally do not account for smoking status (*Coronavirus (COVID-19) Symptom Checker - Check Your Symptoms*, 2023; *COVID-19 Symptom Checker | VCU Health*, 2023; *Symptom Checker*, 2023; *Symptom Checker - St. Luke's's,* 2023). However, our research suggests that 1) mild and moderate symptoms were more commonly reported in people actively using nicotine and/or cannabis compared to non-users, and 2) people actively vaping or dually smoking and vaping tobacco or cannabis products reported more dyspnea than non-users even after adjusting for age, race, comorbidities and other factors. These findings suggest that the use of vaporizers for tobacco or cannabis may not be associated with a difference in self-reported respiratory symptoms compared to smoking tobacco. These findings suggest that smoking and vaping status should be included with other comorbidities in respiratory illness symptom checkers and triage tools, both because it provides context for the symptoms reported, and because it may be a predictor of disease severity. COVID-19 is one of many global respiratory virus outbreaks over the past few decades, and the emergence of novel respiratory virus pandemics in the future is inevitable if historic trends continue (Noor and Maniha, 2020). If smoking and vaping measures are added to symptom checkers, future studies could further address the interplay between smoking and vaporizer use status and symptom reports, and further address associations between product use and clinical outcomes.

Importantly, we found increased respiratory symptoms not just among those smoking cigarettes, but also those using e-cigarettes, smoking cannabis and using cannabis vaporizers. While patients often perceive they may be mitigating the risk of adverse health outcomes by using vaporizers instead of smoking, the patterns of symptoms observed in this study, including severe symptoms, were similar between smoking and vaporizer users. There have been some studies suggesting a protective effect of active smoking for COVID-19 infection (Prinelli et al., 2021). However, this association may be spurious if people who smoke have more symptoms leading to higher rates of COVID-19 testing, and thus a larger proportion of negative tests. The high rates of symptoms among people who smoke and vape in this study are consistent with that explanation. The comorbidities associated with the reporting of potentially more severe symptoms in this study are consistent with previously published literature (Grasselli et al., 2020; Mahamat-Saleh et al., 2021; Richardson et al., 2020), including finding that people who smoke tend to have potentially more severe upper respiratory infections and worse outcomes (Benowitz et al., 2022; Jiang et al., 2020). This study also found that symptoms were common among users of all forms of nicotine and cannabis. Users may perceive that they are decreasing potential health risks by using cannabis instead of nicotine, or by vaping instead of smoking (Bhatia et al., 2022). However, this study shows a similar symptom distribution among all tobacco and cannabis users, and an increased association with dyspnea, which may be a marker of more potentially severe respiratory illness.

This study has several limitations. The COVID-19 & Flu Symptom Checker does not ask respondents about the frequency of use of nicotine and cannabis smoking and vaporized products, so we could not establish whether a dose-dependent association between smoking status and symptoms existed. In addition, the Symptom Checker does not assess for smoking history (i.e. pack-years of smoking), or recent smoking, and only was worded in present tense for “current smoking” without a specific time frame which could lead to misclassification. Since people who are vaping have possibly switched from cigarettes, there may be residual confounding from prior smoking history. Future studies may address this limitation with inclusion of a detailed smoking and vaping history built in as part of the triage tool for better sensitivity. The cross-sectional nature of this study also may not account for an individual's full symptom profile or disease severity, as respondents may have developed further symptoms after use of this triage tool. Both modes of use and type of products are potentially associated with respiratory outcomes, which were separated in our analyses (Bhat et al., 2023). Additionally, we used the reported symptom of dyspnea as a proxy for severity, rather than outcomes like hospitalization or death, because those outcomes were not available in our data set. Because this study was conducted somewhat early in the pandemic, most respondents were unvaccinated. Therefore, it is unknown whether the interaction between smoking status and respiratory viral symptoms would be different in the current environment. Our analytic sample was taken from a patient population which excluded those with who had unique clinical circumstances or reported emergent symptoms requiring immediate medical evaluation, with a prevalence of tobacco use about 7%, lower than the national estimates of ∼20%, but fairly consistent with California rates of cigarette smoking (6.3%) and vaping (3.5%) in 2020–2021 (California Department of Public Health, 2023). Differences due to self-selection of patients into the UCSF symptom checker tool or our patient population may result in decreased generalizability to other parts of the United States. Additionally, this study only includes adult participants, so may not be generalizable to pediatric populations. Lastly, given that all measures were self-reported in the tool, our results may be affected by measurement or recall bias, such as undiagnosed comorbidities. While many studies rely on self-reported measures of tobacco use with high reliability, validating self-reports on symptom checkers with EHR records may increase methodological rigor, reduce bias, and improve reliability in real-world practice (Morales et al., 2013).

## 5 Conclusion

In this study, adults who actively smoked or vaporized nicotine or cannabis products had a higher frequency of upper respiratory illness-related symptoms and an increased association of reporting dyspnea, independent of medical comorbidities. This finding supports considering the inclusion of smoking status in triage questions and symptom checker algorithms for respiratory illnesses. We also found that those reporting nicotine/cannabis vaporizer use had higher rates of dyspnea – a marker of potentially more severe respiratory illness – suggesting there may be detrimental health effects of any type of cannabis or nicotine use.

## CRediT authorship contribution statement

**Sifon U. Ndon:** Writing – review & editing, Writing – original draft, Methodology, Investigation, Formal analysis. **Andrew W. Liu:** Writing – review & editing, Methodology, Investigation, Formal analysis, Data curation. **Timothy J. Judson:** Writing – review & editing, Supervision, Resources, Project administration, Methodology, Data curation, Conceptualization. **Pamela M. Ling:** Writing – review & editing, Supervision, Methodology, Investigation, Conceptualization.

## Declaration of competing interest

The authors declare that they have no known competing financial interests or personal relationships that could have appeared to influence the work reported in this paper.

## Data Availability

The authors do not have permission to share data.

## Appendix A Supplementary data

Supplementary material Supplementary Table 1 (Univariate analyses) and Supplementary Table 2 (Sensitivity analysis). Supplementary material
