# Supplementary material for: COVID-19 symptoms associated with smoking and vaping tobacco and cannabis: A cross-sectional analysis
Source: Prev Med Rep. 2025 Oct 30;60:103291. doi: 10.1016/j.pmedr.2025.103291 (PMC12639576; doi:10.1016/j.pmedr.2025.103291)
Supplement: Supplementary material — Supplementary Table 1 (Univariate analyses) and Supplementary Table 2 (Sensitivity analysis). [file mmc1.docx]

Supplementary Table 1: Univariate associations between mode of tobacco or cannabis product use and dyspnea (n=10,670).

| **Characteristic** | **OR** | **95% CI** |
| --- | --- | --- |
| Smoking or Vaping Tobacco or Cannabis products (vs non-User) |  |  |
| Current Exclusive Smoking | 1.39 | 1.03, 1.85 |
| Current Exclusive Vaporizer Use | 1.74 | 1.14, 2.57 |
| Current Smoking and Vaping | 2.10 | 1.34, 3.18 |

Supplementary Table 2: Sensitivity Analysis of product type associated with dyspnea in multivariable logistic regression controlling for demographics, and comorbidities, excluding patients with chronic lung disease (n=10,670)

| **Characteristic** | **aOR** | **95% CI** |
| --- | --- | --- |
| Active Product Status (vs Non-User) |  |  |
| Current Exclusive Nicotine use | 1.33 | 0.97, 1.78 |
| Current Exclusive Cannabis use | 1.85 | 1.21, 2.75 |
| Current Nicotine and Cannabis use | 2.11 | 1.33, 3.24 |
